# Bailout Stenting After Drug-Coated Balloon Angioplasty: Clinical Outcomes and Implications for Contemporary PCI—A Systematic Review and Meta-Analysis

**DOI:** 10.3390/medicina62091765

**Published:** 2026-09-14

**Authors:** Marcello Marchetta, Branislav Crnomarković, Lucio Giuseppe Granata, Giuseppe Massimo Sangiorgi, Mila Kovačević

**Affiliations:** 1 Institute for Cardiovascular Diseases of Vojvodina, 21204 Sremska Kamenica, Serbia; branislavc89@gmail.com; 2 Faculty of Medicine, University of Novi Sad, 21000 Novi Sad, Serbia; 3 Cardiology Department, Garibaldi-Nesima Hospital, 95122 Catania, Italy; dr.granatacardiologo@gmail.com; 4 Tor Vergata Hospital, 00133 Rome, Italy; gsangiorgi@gmail.com

**Keywords:** drug-coated balloon, bailout stenting, percutaneous coronary intervention, meta-analysis, coronary artery disease

## Abstract

*Background and Objectives*: Drug-coated balloon (DCB) angioplasty represents an established stentless strategy in percutaneous coronary intervention (PCI), although bailout stenting (BOS) is frequently required in the presence of suboptimal angiographic results. The clinical impact of BOS following DCB remains incompletely defined. *Materials and Methods*: We performed a systematic review and single-arm meta-analysis of studies reporting clinical outcomes after BOS following DCB angioplasty, with quantitative synthesis restricted to studies reporting BOS-specific clinical outcomes. Pooled event rates for cardiac death, myocardial infarction (MI), target vessel revascularization (TVR), and target lesion revascularization (TLR) were estimated using random-effects models. Heterogeneity was assessed using standard heterogeneity statistics. *Results*: Five studies were included in the systematic review; four studies reporting BOS-specific clinical outcomes contributed to the quantitative synthesis, whereas REC-CAGEFREE I was retained as strategy-level supportive evidence. BOS after DCB angioplasty was associated with low pooled rates of cardiac death (1%), MI (3%), TLR (5%) and TVR (5%), with minimal or moderate heterogeneity across analyses. Overall, pooled outcomes were within the range reported in contemporary drug-eluting stent (DES) trials. *Conclusions*: BOS following DCB angioplasty was associated with generally low short- to mid-term event rates that were broadly consistent with selected historical DES benchmarks. However, because these comparisons are indirect, comparable safety or efficacy cannot be inferred. BOS may therefore be considered a pragmatic contingency within DCB-based strategies rather than necessarily representing procedural failure.

## 1. Introduction

Drug-coated balloons (DCBs) have emerged as an established stentless strategy in percutaneous coronary intervention (PCI), enabling local delivery of antiproliferative drugs without permanent metallic implantation [1,2]. This “leave nothing behind” approach may better preserve native vessel physiology while avoiding some of the long-term limitations associated with permanent stent implantation, including chronic inflammation, neoatherosclerosis, and very late thrombotic events [3,4]. Clinical evidence has supported the use of DCBs in selected coronary scenarios, particularly in-stent restenosis, small-vessel disease, bifurcation lesions, and clinical settings in which device minimization or shorter dual-antiplatelet therapy (DAPT) may be desirable [5,6,7,8,9]. Randomized controlled trials (RCTs) and large registries have consistently demonstrated the safety and efficacy of DCBs in selected lesion subsets, with outcomes often comparable to contemporary drug-eluting stents (DESs). However, the real-world application of DCBs is frequently limited by suboptimal angiographic results, including flow-limiting dissections, residual stenosis > 30%, and significant vessel recoil. In these scenarios, bailout stenting (BOS) with DESs is frequently required to restore and maintain adequate vessel patency [10]. Contemporary data indicate that BOS is required in approximately 5% to 20% of cases, depending on patient selection, lesion complexity, and procedural technique [11]. Although BOS has traditionally been perceived as a procedural failure within “leave nothing behind” strategies, recent consensus documents and strategy-oriented studies have reframed it as a pragmatic and integral component of contemporary PCI algorithms [10]. Studies such as REC-CAGEFREE I and the DCB-ARC consensus further support BOS as an acceptable and often necessary component of DCB-based strategies, particularly when applied provisionally after adequate lesion preparation [10,12]. However, the safety and prognostic implications of BOS following DCB angioplasty remain poorly defined. In addition, concerns have been raised regarding potential biological interactions between the drug delivered by DCBs and the subsequently implanted stent, particularly in relation to endothelial healing and thrombogenicity [13]. Although multiple observational studies have reported acceptable short- and mid-term outcomes after BOS, their limited sample size, heterogeneity in definitions, and absence of randomized comparisons with DES-only strategies hinder a definitive interpretation. In this context, we performed a systematic review and single-arm meta-analysis to quantitatively assess the incidence of adverse cardiovascular outcomes following BOS after DCB angioplasty. This analysis aims to provide a clinically meaningful estimate of the safety profile of BOS, thereby informing interventional decision making and guiding future research in DCB-based strategies.

## 2. Materials and Methods

### 2.1. Protocol and Registration

This systematic review and meta-analysis was conducted in accordance with the Preferred Reporting Items for Systematic Reviews and Meta-Analysis (PRISMA) 2020 guidelines [14] and the recommendations of the Cochrane Collaboration Handbook for Systematic Reviews of Interventions [15]. The comprehensive PRISMA checklist is provided in File S1. The study protocol was registered in the International Prospective Register of Systematic Reviews (PROSPERO) with the following registration ID: CRD420251078777.

### 2.2. Eligibility Criteria

Studies were considered eligible for the systematic review if they evaluated DCB-based PCI and provided data on bailout stenting (BOS) with DES following suboptimal angiographic results, defined as flow-limiting dissection, significant vessel recoil, residual stenosis > 30%, or TIMI flow < 3. We included prospective and retrospective observational studies as well as RCTs. For the quantitative synthesis, only studies reporting clinical outcomes separately for patients undergoing BOS after DCB angioplasty were included. Studies evaluating a DCB strategy incorporating protocol-defined provisional BOS but reporting clinical outcomes only for the overall treatment arm were retained in the qualitative synthesis as strategy-level supportive evidence. Studies were excluded if they: (1) included overlapping populations; (2) were conference abstracts without full-text peer-reviewed data; or (3) did not provide relevant clinical or procedural data regarding bailout stenting after DCB angioplasty.

### 2.3. Information Sources and Search Strategy

A comprehensive literature search was conducted using PubMed (MEDLINE), Embase, Cochrane Central Register of Controlled Trials (CENTRAL), and Scopus from inception to April 2026. No language or time restrictions were applied. Additional references were identified by manually screening the bibliographies of included studies and through backward and forward citation tracking. The search strategy combined terms related to DCB therapy with terms related to bailout or rescue stenting and suboptimal angiographic results. The complete database-specific search strategies for PubMed, Embase, Scopus, and Cochrane CENTRAL are provided in Appendix A Table A1.

### 2.4. Selection Process

Two reviewers (M.M. and B.C.) independently screened titles and abstracts. Full texts of potentially relevant studies were then retrieved and assessed independently. Discrepancies were resolved through consensus among authors. Zotero was used for reference management, and Rayyan facilitated the screening process. The selection process was documented in sufficient detail to allow for the construction of a PRISMA 2020 flow diagram [14].

### 2.5. Data Collection Process

Two reviewers independently (M.M. and L.G.G.) extracted data using a standardized data collection form. Discrepancies were resolved through discussion. For studies containing both DCB-only and BOS populations, BOS-specific data were extracted whenever separately reported. Studies in which clinical outcomes were available only for the overall DCB-strategy arm were retained for qualitative assessment but did not contribute to the quantitative synthesis.

### 2.6. Data Items

Extracted variables included study design, publication year, sample size, baseline patient characteristics, procedural details, incidence of BOS, and clinical outcomes including cardiac death, myocardial infarction (MI), target lesion revascularization (TLR), and target vessel revascularization (TVR).

### 2.7. Risk of Bias Assessment

Risk of bias was independently assessed by two reviewers (M.M. and B.C.) using the ROBINS-I tool for observational studies [16] and the Cochrane Risk of Bias tool for RCTs [17]. Disagreements were resolved by consensus.

### 2.8. Synthesis Method

For each outcome, the proportion of events reported at the study-defined follow-up was extracted and pooled using random-effects meta-analysis. The primary quantitative synthesis was restricted to studies reporting clinical outcomes specifically for patients undergoing BOS. Because BOS-specific outcomes were not consistently available at a uniform time point across studies, the pooled estimates represent cumulative event rates over the reported short- to mid-term follow-up rather than fixed-time risks. Effect estimates were reported as pooled proportions with 95% confidence intervals (CIs).

Given the absence of a consistent and comparable control group across the available BOS-specific studies, a single-arm meta-analytic approach was adopted to estimate pooled event rates. Studies reporting outcomes only for an overall DCB-strategy population were not included in these pooled estimates. To contextualize these findings, results were descriptively compared with outcomes reported in contemporary benchmark trials evaluating second- and third-generation DESs. Meta-analyses were performed using the metaprop() function from the meta package in R, executed within RStudio (version 2024.12.1). Pooled proportions were calculated using a random-effects model (DerSimonian-Laird estimator) with Wald-type CIs. The Freeman–Tukey double arcsine transformation was applied to stabilize variance, particularly in the presence of rare events and proportions close to 0 or 1. Zero-event studies were handled using the continuity correction implemented in the meta package. Between-study heterogeneity was assessed using Cochran’s Q test, the I^2^ statistic, and τ^2^. An I^2^ value > 50% was considered indicative of substantial heterogeneity.

### 2.9. Reporting Bias Assessment

Contour-enhanced funnel plots were generated for exploratory visualization of potential small-study effects. Because fewer than ten studies contributed to each outcome, funnel plot asymmetry was considered unreliable, and no formal statistical test for asymmetry, such as Egger’s regression, was performed. Accordingly, these plots were not used to infer the presence or absence of publication bias.

## 3. Results

### 3.1. Study Selection

**Figure 1:**
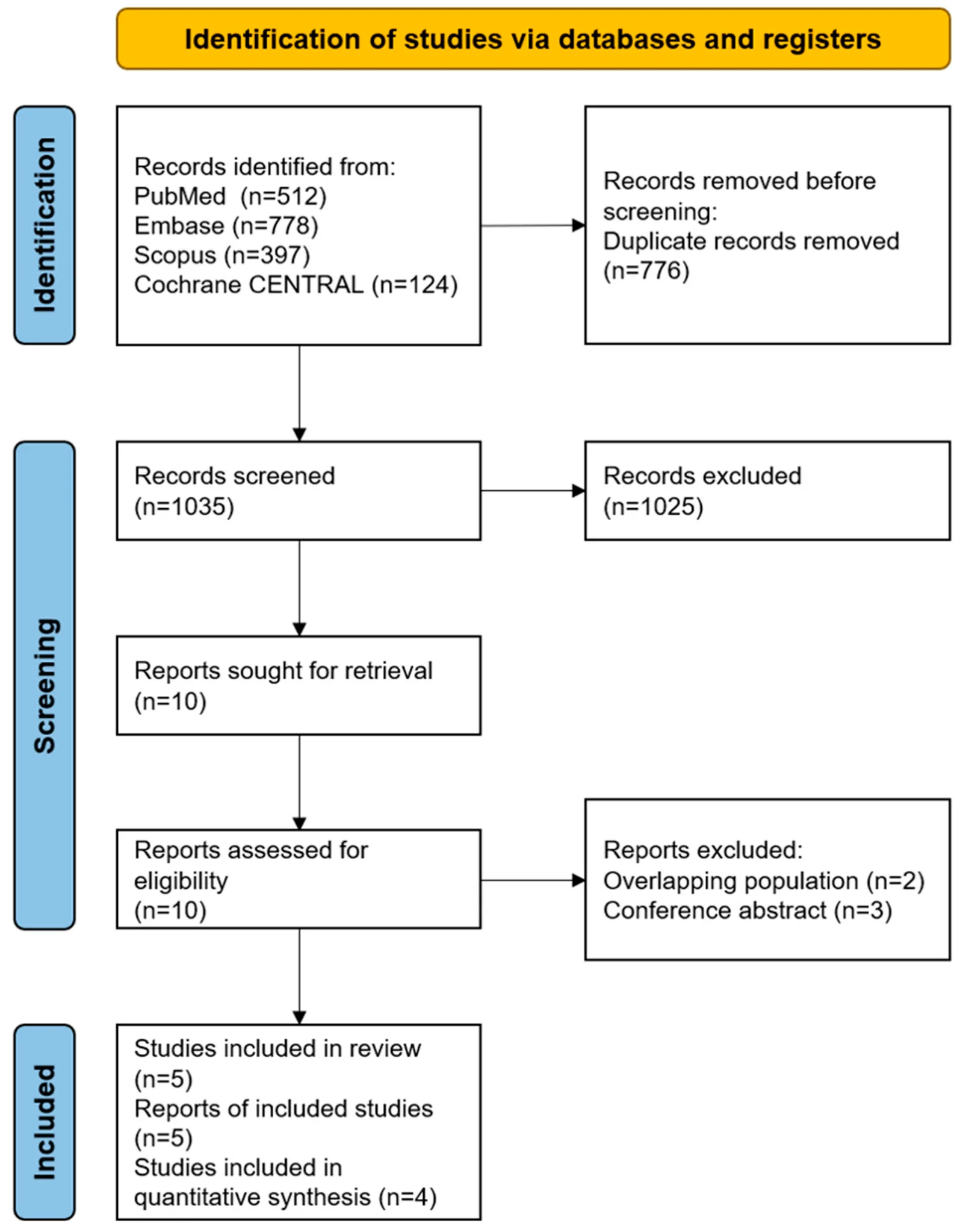
PRISMA 2020 flow diagram of study selection. Flow diagram illustrating process of study identification, screening, eligibility assessment, and inclusion in systematic review and meta-analysis.

A total of 1811 records were identified through database searching (PubMed = 512, Embase = 778, Scopus = 397, Cochrane CENTRAL = 124). After removal of 776 duplicates, 1035 records were screened based on titles and abstracts. Of these, 10 full-text articles were assessed for eligibility. Five studies were excluded due to overlapping populations (*n* = 2) or publication as conference abstracts without full-text-peer-reviewed data (*n* = 3). Ultimately, five studies were included in the systematic review: Gao et al., 2024 [12], Gitto et al., 2026 [18], Gurgoglione et al., 2025 [19], Mitomo et al., 2018 [20], and Mok et al., 2017 [21]. Of these, four studies reporting BOS-specific clinical outcomes were included in the quantitative synthesis, whereas REC-CAGEFREE I (Gao et al. 2024 [12]) was retained as strategy-level supportive evidence and was not included in the pooled analyses. The study selection process is illustrated in Figure 1.

### 3.2. Study Characteristics

Five studies were included in the systematic review. Four observational studies reported BOS-specific clinical outcomes and were included in the quantitative synthesis, whereas REC-CAGEFREE I was retained as strategy-level supportive evidence only. In REC-CAGEFREE I, 106 of 1133 patients allocated to the DCB strategy underwent rescue DES implantation; however, clinical outcomes were reported for the overall DCB-strategy arm and were not separately available for the rescue stenting subgroup. Accordingly, REC-CAGEFREE I did not contribute to any BOS-specific pooled estimate.

The four studies contributing to the quantitative synthesis included dedicated BOS cohorts or separately reported BOS subgroups. The number of patients contributing to individual outcome analyses varied according to endpoint reporting and follow-up availability. Study characteristics are summarized in Table 1.

Baseline patient characteristics were broadly comparable across studies (Table 1), with mean age ranging from 62 to 68 years and a predominance of male patients (63–88%). The prevalence of diabetes ranged from 21% to 48%, while multivessel disease was common when reported.

Clinical and procedural heterogeneity was substantial across the included studies (Table 2 and Table 3). The proportion of de novo lesions ranged from 64.1% to 100%, whereas ISR accounted for 0% to 35.9% of treated lesions. Three studies used paclitaxel-coated balloons exclusively, one used sirolimus-coated balloons exclusively, and one included both technologies. BOS was primarily performed for flow-limiting or advanced dissections and/or significant residual stenosis. Intravascular imaging use was inconsistently reported and, when available, ranged from approximately 9% to 34%; importantly, post-stenting IVUS/OCT use was rarely specified. Follow-up duration ranged from 12 months to approximately 2.4 years; accordingly, the pooled estimates should be interpreted as study-defined short- to mid-term cumulative event rates rather than uniform fixed-time risks.

### 3.3. Risk of Bias

Risk of bias varied across studies. The randomized trial showed low risk of bias with some concerns related to its open-label design. Among observational studies, overall risk of bias ranged from moderate to serious, primarily due to confounding, a lack of standardized treatment strategies, and the absence of independent event adjudication in some cohorts. Detailed risk of bias assessments are provided in Appendix A Table A2 and Table A3.

### 3.4. Quantitative Synthesis

**Figure 2:**
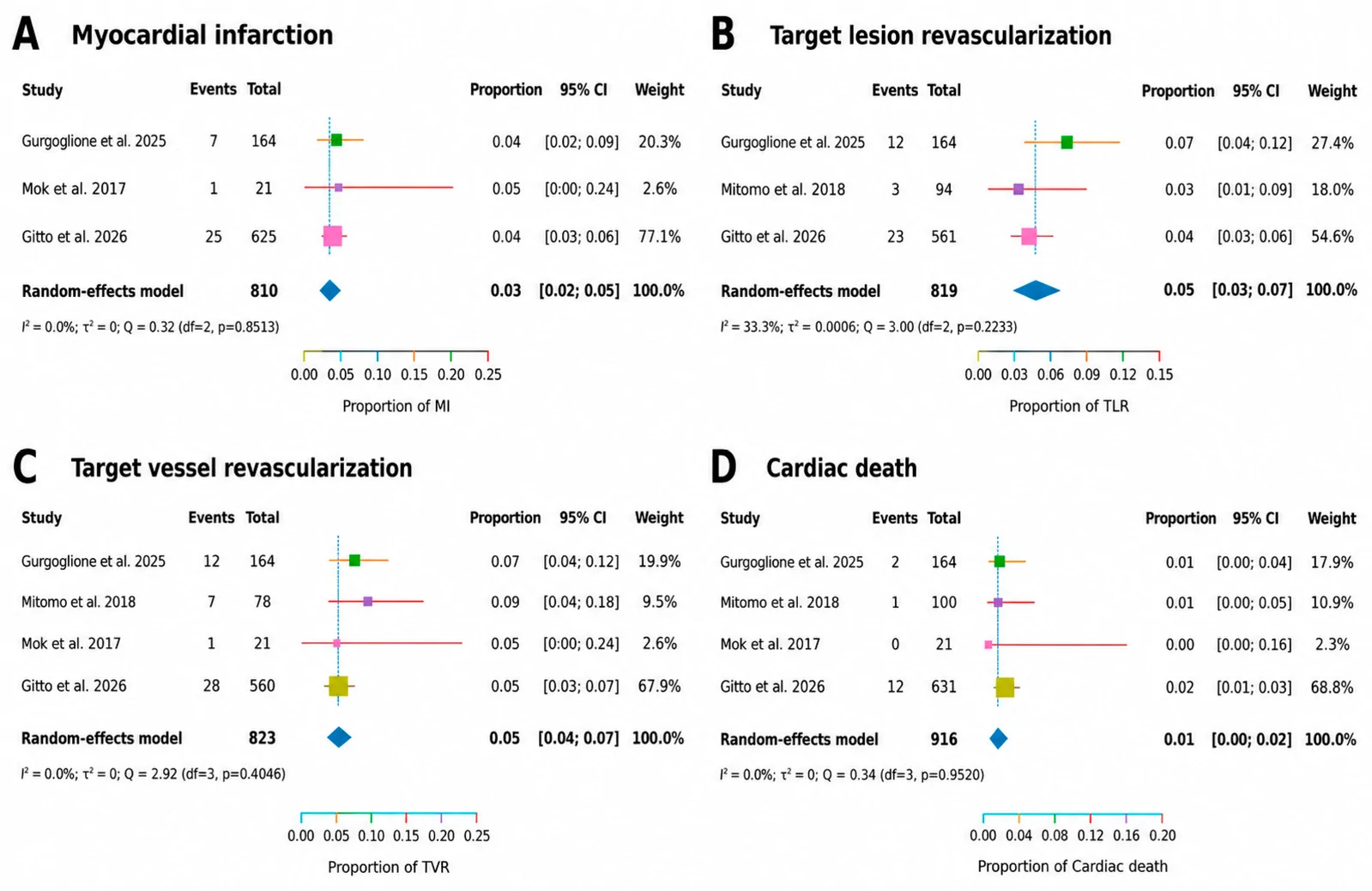
Forest plots of pooled outcome proportions. (**A**) Myocardial infarction; (**B**) target lesion revascularization; (**C**) target vessel revascularization; (**D**) cardiac death. Squares represent study-specific proportions, with size proportional to study weight; horizontal lines indicate 95% confidence intervals; diamonds represent pooled random-effects estimates. The total denominator differs across outcomes because not all studies contributed data to every endpoint and because outcome-specific follow-up availability varied [18,19,20,21].

Quantitative synthesis was performed for four clinical outcomes: cardiac death, MI, TVR, and TLR, including between three and four studies per analysis (Figure 2).

The pooled proportion of cardiac death was 0.01 (95% CI 0.00–0.02), with no evidence of heterogeneity (I^2^ = 0.0%). The pooled proportion of MI was 0.03 (95% CI 0.02–0.05), with no evidence of heterogeneity (I^2^ = 0.0%). The pooled proportion of TVR was 0.05 (95% CI 0.04–0.07), with no evidence of heterogeneity (I^2^ = 0.0%). The pooled proportion of TLR was 0.05 (95% CI 0.03–0.07), with low-to-moderate heterogeneity (I^2^ = 33.3%).

### 3.5. Publication Bias

**Figure A1:**
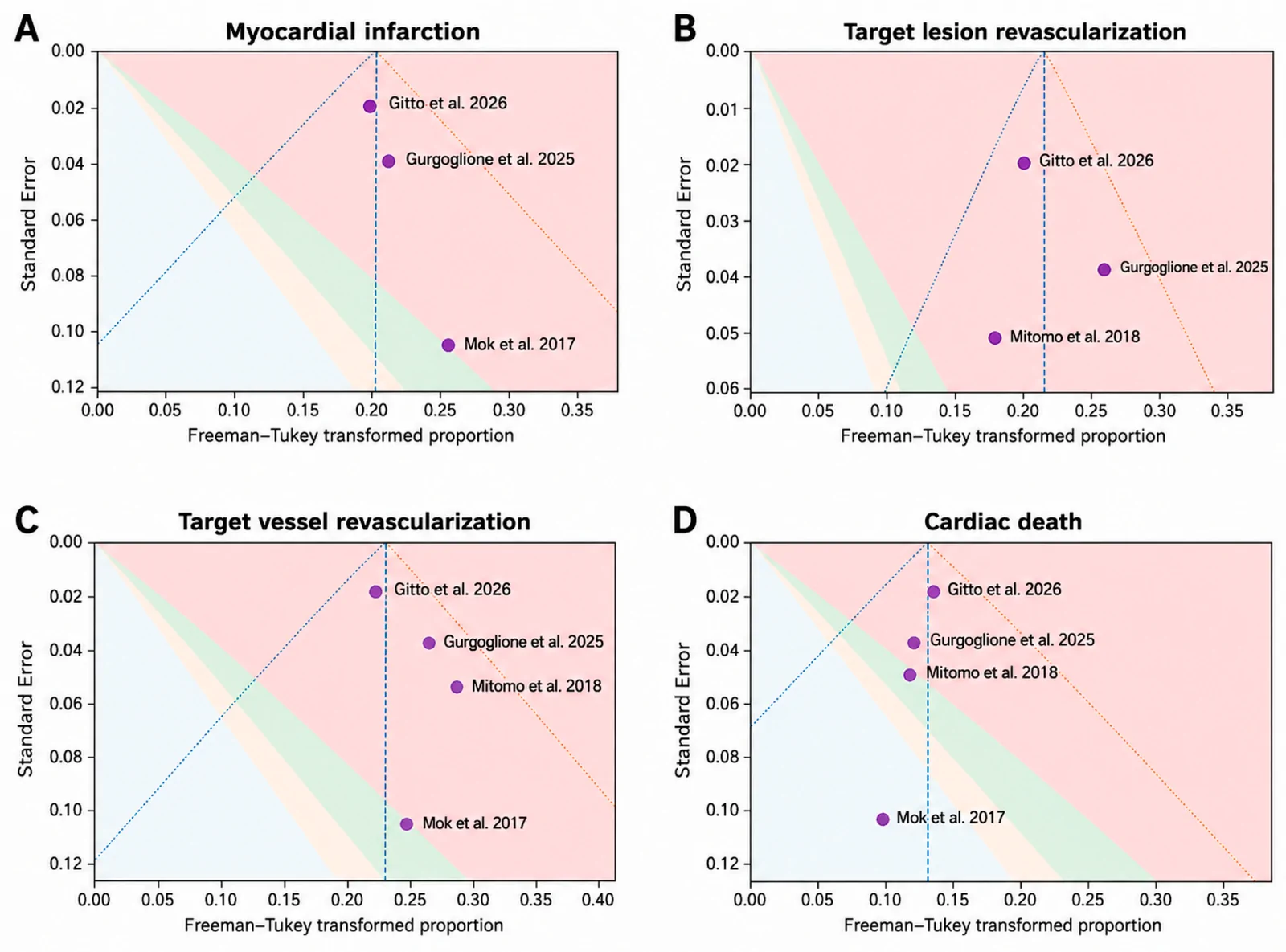
Contour-enhanced funnel plots evaluating potential small-study effects and publication bias. Funnel plots are shown for (**A**) myocardial infarction, (**B**) target lesion revascularization, (**C**) target vessel revascularization, and (**D**) cardiac death. Study-specific Freeman–Tukey-transformed proportions are plotted against their standard errors. The central vertical line denotes the pooled random-effects estimate, and the diagonal lines represent the pseudo 95% confidence limits. Contour shading indicates levels of statistical significance [18,19,20,21].

Contour-enhanced funnel plots for cardiac death, MI, TLR, and TVR are shown in Appendix A Figure A1. Visual inspection did not reveal a consistent pattern of asymmetry across outcomes, although interpretation is limited by the small number of studies contributing to each analysis.

## 4. Discussion

The present study provides the first focused quantitative synthesis of clinical outcomes following BOS after DCB angioplasty in coronary artery disease. The main findings are as follows: (1) BOS after DCB angioplasty is associated with low rates of cardiac death (1%), MI (3%), TLR (5%) and TVR (5%); (2) between-study heterogeneity was negligible for cardiac death, MI, and TVR and remained limited for TLR; and (3) pooled outcomes remain within the range reported in contemporary DES trials. These findings challenge the traditional interpretation of BOS as procedural failure and instead support its role as a predefined contingency within DCB-based PCI strategies. A central conceptual implication of this analysis is that BOS should not be regarded simply as a procedural failure of a “leave nothing behind” strategy. That interpretation is overly simplistic and no longer reflects contemporary DCB practice. In real-world PCI, DCB treatment is inherently conditional on achieving an adequate mechanical result after lesion preparation [22,23]. When this prerequisite is not met because of significant recoil, severe residual stenosis, or flow-limiting dissection, BOS represents a planned procedural safeguard embedded within the DCB strategy rather than a collapse of the strategy itself. Our findings support this view by showing that the occurrence of BOS does not appear to translate into prohibitive short- to mid-term adverse outcomes. In other words, the need to convert to stenting may negate the original “stentless” intention, but it does not necessarily negate the overall safety of the PCI strategy. This distinction is clinically important because it reframes BOS from a binary marker of technical failure into a pragmatic component of lesion-specific decision making. In a BOS model, bailout stenting occurred in 14.5% of de novo lesions treated with DCB-only PCI, and its occurrence was not random, but associated with identifiable clinical and angiographic predictors including prior coronary artery bypass grafting, proximal lesion location, and diffuse disease. The resulting score showed moderate discrimination and excellent calibration after internal validation [24]. That study is highly relevant conceptually because it reinforces the idea that bailout stenting is an anticipated and, to some extent, predictable procedural contingency within DCB programs, particularly in anatomically complex lesions. In that context, bailout is not an aberration but a foreseeable consequence of attempting a stentless strategy in lesions with higher mechanical instability. The mechanistic basis of bailout conversion is also important when interpreting our pooled results. In DCB PCI, the pharmacologic effect of drug delivery does not compensate for an inadequate acute mechanical result [25]. Unlike DES implantation, where scaffolding and drug delivery occur simultaneously, DCB angioplasty relies on lesion preparation to create a stable lumen before drug transfer. This explains why BOS is usually triggered by acute procedural phenomena rather than late biological failure. A recent registry is particularly informative in this regard. In that study, angiographically evident dissections occurred in 39.1% of DCB-treated de novo lesions, but only 21.1% of these dissections required bailout stenting, and untreated, non-flow-limiting dissections were not associated with worse 2-year target lesion failure than lesions without dissection. Moreover, the need for bailout increased with dissection severity, and decision making was guided by angiographic markers of instability such as TIMI flow impairment, persistent extraluminal contrast hang-up, and relevant residual lumen compromise [11]. These observations strongly suggest that the indication for bailout is driven less by the mere presence of vessel injury and more by the presence of acute hemodynamic or structural instability. This point has direct implications for our findings. If BOS is performed selectively in lesions with clinically meaningful procedural instability, then the relatively favorable pooled outcomes observed in our analysis may reflect the effectiveness of bailout conversion itself in neutralizing the immediate hazard created by suboptimal lesion preparation. Put differently, the low pooled rates of cardiac death and MI observed here are consistent with the notion that timely conversion to DES, when angiographically indicated, successfully restores procedural safety. Consistent with this interpretation, the pooled rates of cardiac death and MI were low and showed no evidence of between-study heterogeneity. Another key element of this study is the contextual comparison with contemporary DES outcomes. We deliberately adopted a single-arm meta-analytic model because the available bailout stenting literature lacks a consistent, homogeneous comparator arm. However, contextual benchmarking remains clinically informative. In modern DES trials, event rates for device-oriented outcomes have become progressively lower, and the best contemporary platforms are characterized by low frequencies of cardiac death, TVR, repeat revascularization, and stent thrombosis (Table 4). In BIOFLOW V, 12-month target lesion failure occurred in 6% of patients treated with a bioresorbable-polymer sirolimus-eluting stent and 10% of those treated with a durable-polymer everolimus-eluting stent, while cardiac death remained below 1% and clinically driven target lesion revascularization around 2% [26]. In EVOLVE II, 12-month TVR was 3.8% versus 3.6%, TLR 2.6% versus 1.7%, and cardiac death 0.5% versus 0.9% across the two DES platforms, with definite/probable stent thrombosis below 1% [27]. At longer follow-up, BIOSCIENCE reported similar 5-year outcomes between biodegradable-polymer sirolimus-eluting and durable-polymer everolimus-eluting stents, with TLR around 10%, TVR around 13.5%, and definite stent thrombosis 1.6% in both groups [28]. Consistently, long-term data from trials such as ISAR-TEST 5 have shown that adverse events continue to accumulate over time, largely reflecting disease progression rather than device failure [29]. Similarly, in large-scale all-comer PCI trials such as GLOBAL LEADERS, rates of death and myocardial infarction at 2-year follow-up remained low but non-negligible, reflecting the combined influence of device performance and underlying patient risk rather than procedural factors alone [30]. Against this background, the pooled event rates observed after BOS fell within the broad numerical range reported in selected contemporary DES trials. However, these comparisons are indirect and descriptive. Differences in patient characteristics, lesion complexity, procedural strategies, follow-up duration, and endpoint definitions preclude any inference of equivalence, non-inferiority, or comparable safety. The DES data should therefore be considered only as historical clinical benchmarks rather than as a formal comparator. REC-CAGEFREE I warrants separate consideration as strategy-level supportive evidence. In this randomized trial, 106 of 1133 patients assigned to the DCB strategy underwent rescue DES implantation after an unsatisfactory angiographic result; however, clinical outcomes were reported for the overall DCB-strategy arm rather than separately for the rescue-stenting subgroup [12]. Accordingly, REC-CAGEFREE I was not included in the BOS-specific quantitative synthesis. Nevertheless, the trial remains clinically relevant because it provides randomized evidence on the feasibility and limitations of a DCB-first strategy incorporating protocol-defined provisional rescue stenting. From a clinical standpoint, our findings support a more nuanced message for interventional practice. DCB programs should not be judged solely by the proportion of cases completed without stent implantation. A more meaningful assessment is whether operators can achieve low adverse-event rates while using a disciplined strategy for lesion preparation, careful angiographic assessment, and low-threshold conversion in the presence of instability. Under that paradigm, BOS becomes part of a quality-controlled DCB workflow. Indeed, the emerging literature supports structured decision making rather than rigid adherence to a stentless dogma. The work by Iossa et al. suggests that preprocedural identification of lesions at higher bailout risk may improve planning and patient selection [23], whereas the study by Gitto et al. indicates that non-flow-limiting dissections can often be managed conservatively without compromising outcomes when objective angiographic criteria are satisfied [30]. Together with our pooled data, these observations support a strategy in which DCB angioplasty and BOS are viewed as complementary procedural tools rather than mutually exclusive philosophies. These considerations are especially relevant as DCB use expands beyond ISR and small-vessel disease into more complex de novo settings. As the treated anatomy becomes more proximal, diffuse, calcified, or bifurcational, the probability of acute mechanical imperfection increases, and so does the relevance of a bailout framework. The concern in such settings is not merely whether bailout occurs, but whether bailout, once required, compromises prognosis. Our data suggest that it does not do so to a clinically alarming extent. This does not imply that all lesions are equally suitable for a DCB-first approach, nor that conversion rates are irrelevant. Rather, it suggests that the safety net of DES remains effective when selectively deployed after DCB preparation. This is an important practical reassurance for operators who may otherwise be reluctant to consider DCB strategies because of the perceived stigma attached to bailout conversion. At the same time, our data should not be used to minimize the importance of lesion selection and procedural technique. The favorable pooled outcomes reported here likely depend on appropriate operator judgment. Inadequate lesion preparation, inaccurate vessel sizing, under-recognition of deep dissection, or delayed conversion in unstable lesions could plausibly worsen outcomes. Moreover, the heterogeneity observed for TLR indicates that procedural and study-level variability still matters.

Future research should therefore focus not only on clinical outcomes after bailout, but also on standardizing the procedural triggers for bailout and refining the tools that guide the decision. Intravascular imaging may be particularly useful by detecting stent underexpansion, malapposition, edge dissection, or geographic miss not apparent on angiography. Because post-stenting IVUS/OCT use was limited or inconsistently reported across the included cohorts, unrecognized suboptimal stent deployment could have contributed to the observed variability in TLR; however, the available aggregate data do not permit this effect to be quantified. Despite these considerations, the findings of this analysis should be interpreted in light of several limitations that should be acknowledged. First, confidence in the pooled estimates is limited by the small evidence base, with only four observational studies contributing BOS-specific clinical outcomes to the quantitative synthesis. Several of these studies had a moderate-to-serious risk of bias. Residual confounding, selection bias, operator-dependent treatment decisions, non-standardized procedural strategies, and incomplete or non-independent event adjudication may therefore have influenced the observed event rates. Accordingly, the pooled estimates should be considered hypothesis-generating and should not be interpreted as definitive evidence of the comparative safety or efficacy of BOS.

Second, the single-arm design, although methodologically justified by the absence of a consistent comparator group, precludes direct causal inference and formal comparison with DES-only strategies. Importantly, no randomized study provided BOS-specific clinical outcomes suitable for quantitative pooling. REC-CAGEFREE I reported outcomes for the overall DCB-strategy arm rather than separately for patients undergoing rescue DES implantation and was therefore considered only as strategy-level supportive evidence. Third, endpoint definitions, follow-up duration, and procedural characteristics were not fully standardized across studies. Follow-up ranged from 12 months to approximately 2.4 years; because cumulative event proportions are time-dependent, pooling estimates reported at different time points may have introduced time-at-risk bias. A dedicated 12-month analysis was not performed because BOS-specific outcomes at this time point were not consistently reported across all included studies, and restricting the synthesis to 12 months would have further reduced the already limited evidence base. Accordingly, the pooled estimates should be interpreted as overall short- to mid-term event rates rather than fixed 12-month risks, particularly for target lesion revascularization. Information on DAPT, including duration and regimen, was not consistently reported and could not be systematically analyzed, despite its potential impact on post-procedural outcomes. Clinical heterogeneity across lesion substrate, DCB platform, imaging guidance, and follow-up duration may have influenced the pooled estimates, particularly for TLR. However, inconsistent reporting and the small number of studies precluded a reliable assessment of the independent contribution of these factors. For the same reasons, subgroup analyses according to lesion type, DCB technology, or study design would have included too few studies per stratum to yield reliable estimates and were therefore not performed. Granular lesion-level characteristics, including calcium burden, dissection morphology, balloon-to-vessel ratio, and lesion preparation techniques, were also incompletely reported, further limiting mechanistic interpretation of the results. Finally, the relatively small number of included studies limits the reliability of publication bias assessment and increases the influence of individual studies on pooled estimates.

## 5. Conclusions

In this systematic review and single-arm meta-analysis, BOS after DCB angioplasty was associated with generally low rates of cardiac death, MI, and repeat revascularization. These findings suggest that BOS may represent a pragmatic contingency after suboptimal DCB results; however, the absence of a direct comparator precludes conclusions regarding excess risk or equivalence relative to DES-only strategies. Given the limited and observational evidence base, these findings should be considered hypothesis-generating. Further prospective studies are warranted to refine bailout criteria and identify the lesion subsets most likely to benefit from a DCB-first approach.

## Figures and Tables

**Table 1:** Baseline characteristics of included studies.

Study	Design	Source Cohort	BOS Subgroup	Age ^†^, y	BMI ^†^, kg/m^2^	Male Sex, %	Follow-Up Duration ^†^, y	Diabetes, %	HTN (%)	Current Smoker, %	Dyslipidemia, %	LVEF ^†^, %	CKD *, %
Gao et al., 2024 * [12]	RCT	1133	106	62.0	24.6	67.8	2	24.8	58.4	31.7	79.6	NA	8.4
Gitto et al., 2026 [18]	Observational, retrospective	733	733	68.0	27.2	82.7	1	26.5	NA	NA	NA	53.8	8.0
Gurgoglione et al., 2025 [19]	Observational, retrospective	2084	164	65.0	27.0	78.0	2	37.2	NA	NA	NA	55.0	8.5
Mitomo et al., 2018 [20]	Observational, retrospective	103	103	68.6	NA	88.3	2.4	21.3	60.2	59.2	76.6	52.6	51.4
Mok et al., 2017 ^A^ [21]	Observational, retrospective	76	76	65.9	NA	63.1	1	48.7	72.4	51.4	NA	47.9	NA

Abbreviations. BMI: body mass index; CKD: chronic kidney disease; HTN: hypertension; LVEF: left ventricular ejection fraction; NA: not available; RCT: randomized controlled trial. ^†^ Mean or median; * Gao et al., 2024 [12] and Mitomo et al., 2018 [20] classified CKD as estimated glomerular filtration rate (eGFR) <60 mL/kg/m^2^, the other studies did not provide any definition of CKD. ^A^ In Mok et al. 2017 [21], although patients were divided into two cohorts based on the type of stent implanted after drug-coated balloon angioplasty (DCB, both groups underwent bailout stenting after suboptimal PCB angioplasty. For the purposes of this single-arm meta-analysis on bailout stenting, the two groups were considered as a single cohort. * In REC-CAGEFREE I, the reported population and clinical outcomes refer to the overall DCB-strategy arm (*n* = 1133), of whom 106 underwent rescue DES implantation; BOS-specific clinical outcomes were not separately reported. Source cohort denotes the population from which data were derived in each included publication, whereas BOS subgroup denotes the number of patients who underwent bailout stenting. Baseline characteristics refer to the BOS subgroup when separately reported and otherwise to the corresponding source cohort.

**Table 2:** Baseline clinical and procedural characteristics of included studies.

Study	Multivessel Disease, %	STEMI, %	NSTEMI, %	UA, %	ISR, %	De Novo Lesion, %	AHA/ACC B2/C Lesion *, %	Recoil/Residual Stenosis **, %	Type C-F Dissection **, %	TIMI Flow < 3 **, %
Gao et al., 2024 [12]	NA	15.9	18.8	20.7	0	100	NA	18	82	NA
Gitto et al., 2026 [18]	24.5	9.5	19.4	8.0	11.5	88.5	73.5	35.5	61.8	2.7
Gurgoglione et al., 2025 [19]	56.7	8	20.7	20.7	35.9	64.1	43.1	NA	NA	NA
Mitomo et al., 2018 [20]	85.4	0.9	0	0	24.8	75.2	NA	63.2	36.8	NA
Mok et al., 2017 ^A^ [21]	61.8	7.9	34.2	34.2	NA	NA	100	51.3	10.5	NA

Abbreviations. AHA/ACC: American Heart Association/American College of Cardiology; ISR: in-stent restenosis; NA: not available; STEMI: ST elevation myocardial infarction; UA: unstable angina. * Type B2 and C lesions are characterized by features such as lesion length > 20 mm, excessive tortuosity, severe calcification, bifurcation involvement, thrombus, or total occlusion. Data refer to the percentage of target lesions meeting these morphological criteria when explicitly reported by the authors. ** Reason for bailout stenting. ^A^ In Mok et al., 2017 [21], although patients were divided into two cohorts based on the type of stent implanted after PCB (bare-metal stent vs. drug-eluting stent), both groups underwent bailout stenting after suboptimal PCB angioplasty. For the purposes of this single-arm meta-analysis on bailout stenting, the two groups were considered a single cohort.

**Table 3:** Procedural and device details of included studies.

Study	Predilatation, %	DCB Paclitaxel, %	DCB, Sirolimus, %	DCB Caliber ^†^, mm	DCB Length ^†^, mm	BO DES Caliber ^†^, mm	BO DES Length ^†^, mm	IVUS/OCT After Stenting
Gao et al., 2024 [12]	100	100	0	NA	NA	NA	NA	NA
Gitto et al., 2026 [18]	93.7	52.7	47.3	2.5	30.0	3.0	28	NA
Gurgoglione et al., 2025 [19]	88.4	NA	NA	2.42	14.60	2.56	21.86	9.6
Mitomo et al., 2018 [20]	NA	100	0	NA	NA	NA	NA	NA
Mok et al., 2017 [21]	100	100	0	2.9	30.67	2.89	28.08	NA

Abbreviations: BO: bailout; DCB: drug-coated balloon; DES: drug-eluting stent; IVUS: intravascular ultrasound; OCT: optical coherence tomography. ^†^ Mean or median.

**Table 4:** Characteristics of benchmark studies.

Study	Year	Clinical Population	Stent Type	*N*	Follow-Up	TLR (%)	TVR (%)	Cardiac Death (%)	MI (%)
EVOLVE II [26]	2015	Selected de novo PCI; stable CAD or NSTE-ACS	BP-EES vs. DP-EES	1684	12 mo	2.6	2.6	0.4–0.6	~3.0
BIOFLOW V [25]	2017	Selected de novo native-vessel PCI; elective or urgent presentation	BP-SES vs. DP-EES	1334	12 mo	4.5–5.1	NA	0.1	~4.0
BIOSCIENCE [27]	2018	All-comer PCI; stable CAD or ACS	BP-SES vs. DP-EES	2119	12 mo	3.5–3.8	3.5–3.8	1.0–1.5	3.0–4.0
ISAR-TEST 5 [28]	2020	Broad CAD population with de novo lesions	PF-DES vs. DP-ZES	3002	10 y	~20	NA	~27	NA
GLOBAL LEADERS [29]	2018	All-comer PCI; stable CAD or ACS	Biolimus DES	15,968	24 mo	NA	NA	2.5–3.0	NA

Abbreviations: ACS: acute coronary syndrome; BP-EES: biodegradable polymer everolimus-eluting stent; BP-SES: biodegradable polymer sirolimus-eluting stent; CAD: coronary artery disease; CD: cardiac death; DES: drug-eluting stent; DP-EES: durable-polymer everolimus-eluting stent; DP-ZES: durable-polymer zotarolimus-eluting stent; MI: myocardial infarction; NA: not available; NSTE-ACS: non-ST elevation acute coronary syndrome; PCI: percutaneous coronary intervention; PF-DES: polymer-free drug-eluting stent; TVF: target vessel failure; TVR: target vessel revascularization.

## Data Availability

The data supporting the findings of this study are available from the corresponding author upon reasonable request.

## Supplementary Materials

The following supporting information can be downloaded at: https://www.mdpi.com/article/10.3390/medicina62091765/s1, File S1: PRISMA 2020 Checklist.

## Abbreviations

The following abbreviations are used in this manuscript:

BOS	Bailout Stenting
CENTRAL	Cochrane Central Register of Controlled Trials
CI	Confidence Interval
DAPT	Dual Antiplatelet Therapy
DCB	Drug-Coated Balloon
DES	Drug-Eluting Stent
MI	Myocardial Infarction
PCI	Percutaneous Coronary Intervention
PRISMA	Preferred Reporting Items for Systematic Reviews and Meta-Analysis
PROSPERO	Prospective Register of Systematic Reviews
RCT	Randomized Controlled Trial
TLR	Target Lesion Revascularization
TVR	Target Vessel Revascularization

## Appendix A

**Table A1:** Detailed search strategy for electronic databases.

PubMed	(“drug coated balloon” OR “drug coated balloons” OR DCB OR DCBs OR “drug eluting balloon” OR “drug eluting balloons” OR DEB OR DEBs OR “paclitaxel coated balloon” OR “sirolimus coated balloon”) AND (“bail-out stenting” OR “bailout stenting” OR “rescue stenting” OR “conversion to stent” OR “stent implantation” OR “unplanned stenting” OR “need for stenting” OR “emergent stenting” OR “bail-out stent” OR “stent conversion” OR “suboptimal angiograph*”)
Embase (via Ovid)	(“drug coated balloon” OR “drug coated balloons” OR DCB OR DCBs OR “drug eluting balloon” OR “drug eluting balloons” OR DEB OR DEBs OR “paclitaxel coated balloon” OR “sirolimus coated balloon”) AND (“bail-out stenting” OR “bailout stenting” OR “rescue stenting” OR “conversion to stent” OR “stent implantation” OR “unplanned stenting” OR “need for stenting” OR “emergent stenting” OR “bail-out stent” OR “stent conversion” OR “suboptimal angiograph*”)
Scopus	(“drug coated balloon” OR “drug coated balloons” OR DCB OR DCBs OR “drug eluting balloon” OR “drug eluting balloons” OR DEB OR DEBs OR “paclitaxel coated balloon” OR “sirolimus coated balloon”) AND (“bail-out stenting” OR “bailout stenting” OR “rescue stenting” OR “conversion to stent” OR “stent implantation” OR “unplanned stenting” OR “need for stenting” OR “emergent stenting” OR “bail-out stent” OR “stent conversion” OR “suboptimal angiograph*”)
Cochrane CENTRAL	(“drug coated balloon” OR “drug coated balloons” OR DCB OR DCBs OR “drug eluting balloon” OR “drug eluting balloons” OR DEB OR DEBs OR “paclitaxel coated balloon” OR “sirolimus coated balloon”) AND (“bail-out stenting” OR “bailout stenting” OR “rescue stenting” OR “conversion to stent” OR “stent implantation” OR “unplanned stenting” OR “need for stenting” OR “emergent stenting” OR “bail-out stent” OR “stent conversion” OR (“suboptimal” NEXT angiograph*)

**Table A2:** Risk of bias summary for randomized studies (RoB 2).

Study	Bias from Randomization Process	Bias Due to Deviations from Intended Interventions	Bias Due to Missing Outcome Data	Bias in Measurement of the Outcomes	Bias in Selection of the Reported Result	Overall Risk of Bias
Gao et al., 2024 [12]	Low	Some concerns	Low	Low	Low	Some concerns

Color legend: green indicates low risk of bias, yellow indicates some concerns.

**Table A3:** Risk of bias summary for non-randomized studies (ROBINS-I).

Study	Bias Due to Confounding	Bias in Selection of Participants	Bias in Classification of Interventions	Bias Due to Deviations from Intended Interventions	Bias Due to Missing Data	Bias in Measurement of Outcomes	Bias in Selection of the Reported Result	Overall Risk of Bias
Gitto et al., 2026 [18]	Moderate	Low	Low	Moderate	Moderate	Serious	Moderate	Serious
Gurgoglione et al., 2025 [19]	Moderate	Low	Low	Moderate	Low	Low	Moderate	Moderate
Mitomo et al., 2018 [20]	Serious	Moderate	Low	Serious	Moderate	Moderate	Moderate	Serious
Mok et al., 2017 [21]	Serious	Moderate	Low	Serious	Moderate	Moderate	Moderate	Serious

Color legend: green indicates low risk of bias, yellow indicates some concerns, and orange indicates high risk of bias.
